# Using the DIVA model to better understand speech motor disorders

**DOI:** 10.1080/02699206.2026.2680130

**Published:** 2026-07-22

**Authors:** Hannah P. Rowe, Frank H. Guenther

**Affiliations:** a Department of Communication Sciences and Disorders, Northeastern University, Boston, Massachusetts, USA;; b Departments of Speech, Language, & Hearing Sciences and Biomedical Engineering, Boston University, Boston, Massachusetts, USA;; c Picower Institute of Learning and Memory, Massachusetts Institute of Technology, Cambridge, Massachusetts, USA

**Keywords:** Neurocomputational modeling, DIVA model, speech motor control, dysarthria, apraxia, stuttering

## Abstract

The Directions Into Velocities of Articulators (DIVA) model provides a comprehensive neurocomputational framework for understanding speech production and speech motor disorders. This theoretical model describes three main control processes: feedforward control, auditory feedback control, and somatosensory feedback control. By mapping these processes to specific brain regions and neural circuits, DIVA offers insights into the mechanistic basis of various speech disorders and provides a foundation for developing targeted therapeutic interventions. This paper reviews the DIVA model’s architecture, its applications to understanding communication disorders, and its potential for guiding clinical practice and technology development.

## Introduction

Understanding how the brain controls speech is key to improving both scientific models and clinical approaches to communication disorders. Several computational models of speech motor control have been proposed to account for how the brain transforms linguistic representations into coordinated articulatory movements. Among these are the State Feedback Control (SFC) model (Houde & Nagarajan, 2011), which emphasises internal forward models and state estimation; the Hierarchical State Feedback Control (HSFC) model (Hickok, 2012, 2014), which integrates psycholinguistic and motor control perspectives at hierarchically organised levels of sensorimotor representation; and task-dynamic approaches (Parrell et al., 2019; Saltzman & Munhall, 1989), which model speech as the coordination of dynamically defined articulatory gestures. Each contributes important insights, and these models share certain features while differing in others (see Guenther & Hickok, 2016 for a detailed comparison).

**Figure 1.**
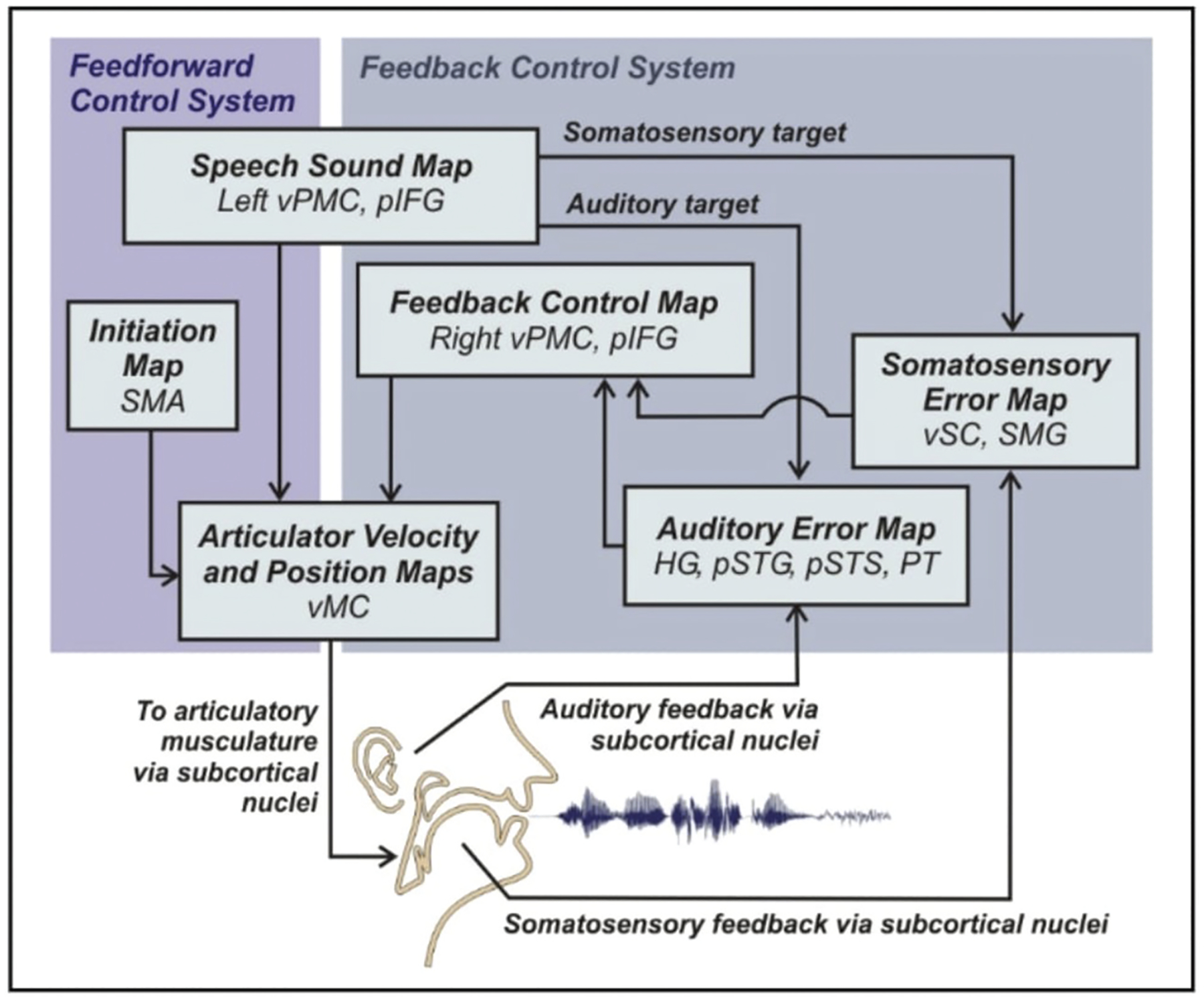
Simplified block diagram of the cortical components of the DIVA model. Boxes = cortical maps of neurons; arrows = synaptic projections; vPMC = ventral premotor cortex; pIFG = posterior inferior frontal gyrus; SMA = supplementary motor area; vMC = ventral motor cortex; vSC = ventral somatosensory cortex; SMG = supramarginal gyrus; HG = Heschl’s gyrus; pSTG = posterior superior temporal gyrus; pSTS = posterior superior temporal sulcus; PT = planum temporale.

The present paper focuses on the Directions Into Velocities of Articulators (DIVA) model (Guenther, 2006, 2016; Tourville & Guenther, 2011), which is schematised in Figure 1. We focus on DIVA for three reasons that make it particularly well suited to the clinical-translational goals of this paper. First, DIVA is the most extensively developed neurocomputational model with explicit mapping of computational processes to specific brain regions and neural circuits, allowing for direct comparison between model predictions and both behavioural and neuroimaging data. Second, DIVA has been directly applied to a broader range of clinical speech motor disorders, including dysarthrias, apraxia of speech, and stuttering (e.g. Civier et al., 2013; Kearney et al., 2020; Terband et al., 2009). Third, the model’s neuroanatomical grounding makes it uniquely suited for bridging from neuroscience to clinical practice, allowing clinicians to link observed speech deficits to specific disruptions in neural circuitry. This focus is not intended to diminish the contributions of other models but rather reflects DIVA’s suitability for the specific aims of the present work.

For researchers, the DIVA model can generate mechanistic, testable predictions to inform experimental design and provide a framework for interpreting data. For clinicians, it can highlight how specific neural systems support different aspects of speech, potentially aiding in the development of targeted interventions that leverage a patient’s preserved motor control mechanisms. This manuscript begins with an overview of the DIVA model’s architecture and functional principles. We then discuss how communication disorders, particularly those involving impaired speech motor control, can be understood within this framework.

## Part I: Overview of the DIVA model

**Figure 2.**
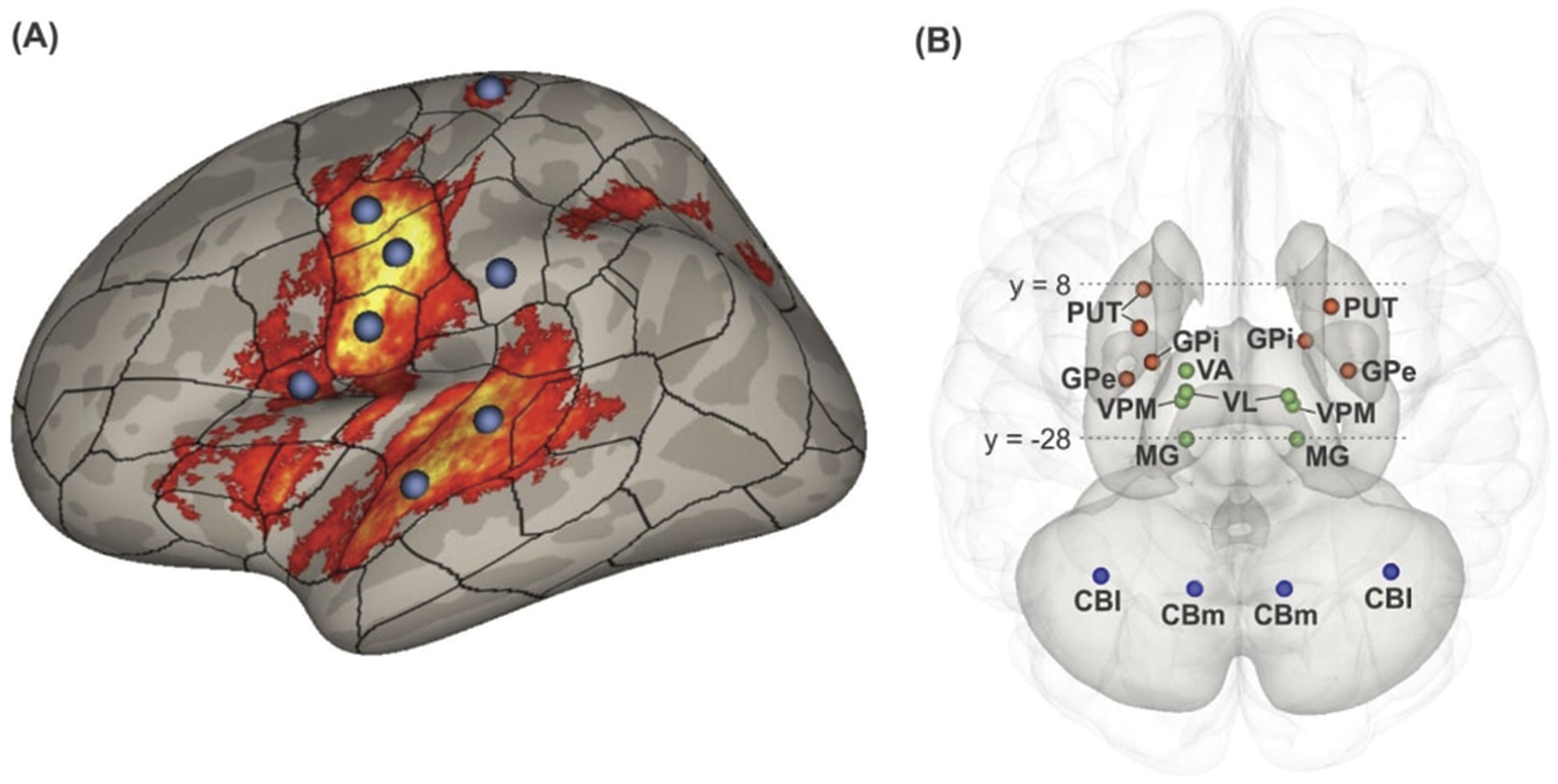
Schematic illustrating how each component of the model is mapped to specific brain regions.

At its core, the DIVA model captures the dynamic interplay between three principal control processes: feedforward control, auditory feedback control, and somatosensory feedback control. Each process plays a unique role in speech production, while working together in an integrated system. Implemented as an adaptive neural network, the DIVA model transforms abstract speech targets (e.g. phonemes, syllables, words) into time-varying articulator movements, which then drive an articulatory synthesiser to produce speech output. Each component of the model (conceptualised as ‘neurons’ or ‘nodes’) is mapped to a specific brain region, making it uniquely suited for bridging behaviour, computation, and brain function (Figure 2). The model provides: (1) a computational account of speech that can be computer-simulated, allowing for direct comparison with acoustic speech data; and (2) a neuroanatomically grounded account of where these computations occur in the brain, allowing for direct comparison with neuroimaging data.

### Feedforward control

Humans are capable of exceptionally rapid and precise articulatory movements. Numerous researchers have argued that the speed of conversational speech is far too great to depend solely on feedback mechanisms, given the inevitable neural delays in processing sensory information (e.g. Lashley, 1951; Lenneberg, 1967; Neilson & Neilson, 1987). To illustrate, a speaker can produce the word ‘dilapidated’ in roughly one second or less, which corresponds to an average movement time of under 100 milliseconds per sound. Given that the auditory feedback system operates with a latency of about 100–150 milliseconds (discussed further in the following section), the full set of muscular commands for a given sound must often be executed before any auditory feedback from the movement can be processed, making exclusive reliance on auditory feedback control impossible. As Lashley (1951) observed, this necessity points to the presence of ‘some central nervous mechanism which fires with predetermined intensity and duration or activates different muscles in predetermined order’ (p. 516). We call this the feedforward control system.

In the DIVA model, feedforward control refers to the generation of stored motor commands (also known as motor programs or gestural scores) that drive the production of speech sounds without continuous monitoring of their sensory consequences. This mode of control, often described as ‘open-loop’, enables rapid and fluent speech by allowing the speaker to initiate articulatory movements based on previously learned patterns rather than on real-time sensory feedback.

Crucially, these motor programs are not innate or hardwired; they are acquired through experience and practice. Because speech production is highly language-specific, each individual must learn and refine motor programs that are appropriate for the sounds and syllables of their native language (Guenther, 2006). In the DIVA framework, this learning occurs through an adaptive process in which corrective commands generated by the auditory and somatosensory feedback systems are incrementally incorporated into the feedforward command. Over repeated attempts at producing a speech sound, the feedforward system gradually internalises these corrections, resulting in more accurate and efficient motor programs for future productions. Take an analogy of driving around a curve on the highway: Feedforward control can be likened to slowing down in anticipation of a curve in the road ahead, as the feedforward system sets you up for your goal (i.e. staying in your lane) based on previously learned patterns that have been gradually updated over time (i.e. slowing down when you see a curve in the road).

This tuning process allows the feedforward controller to become increasingly reliable over time, reducing the system’s dependence on slower feedback mechanisms and enabling the fluent execution of speech. Once fully developed, the feedforward control system serves as the primary driver of speech in adults, accounting for the majority of articulatory movements during normal, unperturbed speaking conditions.

### Feedback control

Complementing feedforward control are two feedback control subsystems: auditory and somatosensory. In our driving analogy, feedback control can be likened to swerving back into the lane after hitting a highway rumble strip, as the feedback system monitors the current state (i.e. driving in the lane) for errors (i.e. driving onto the rumble strip) and adjusts it accordingly (i.e. swerves) to match the desired goal (i.e. staying in your lane). Auditory feedback control operates by comparing the acoustic output of speech to internally specified auditory targets. When deviations occur, the system generates corrective motor commands to reduce the error. However, as aforementioned, this process operates with a latency exceeding 100 milliseconds, which makes it too slow to serve as the primary controller of fluent speech (Guenther et al., 2006; Houde & Nagarajan, 2011). Similarly, somatosensory feedback control compares incoming tactile and proprioceptive input from the articulators to stored somatosensory targets. Though slightly faster (with a latency of approximately 50 milliseconds), it too is primarily useful during learning and for correcting unexpected perturbations (Guenther et al., 2006).

### Learning in the model

The DIVA model also provides a detailed account of how these systems develop through learning. Early in speech development, the model engages in a ‘babbling’ phase, producing random articulatory movements that allow it to map relationships between motor commands and the resulting sensory feedback. These exploratory movements help calibrate both auditory and somatosensory feedback systems. As development progresses, the model refines its control through imitation and repetition. It listens to fluent speech to construct time-varying auditory targets, which are multi-dimensional regions in auditory space that represent acceptable acoustic variability. These targets are encoded in projections from premotor to auditory cortex.

Repeated practice with a given sound allows the model to refine its motor programs by incorporating error corrections from the feedback systems. Importantly, somatosensory targets are not learned until the child successfully produces a sound and experiences what it ‘feels like’ to say it correctly. These targets are learned gradually and continue to adapt as the vocal tract grows, ensuring that the somatosensory feedback system remains aligned with the evolving physical characteristics of the speaker.

As these processes unfold, the interplay between feedforward and feedback control becomes increasingly biased towards feedforward control. In the early stages of learning, feedforward commands are imprecise, necessitating strong contributions from the feedback systems to maintain intelligibility. However, as the feedforward controller incorporates these corrections, the need for feedback control diminishes. Ultimately, in fluent adult speech, the majority of productions are governed by well-tuned feedforward commands, with feedback control acting primarily as a fine-tuning mechanism, though remains capable of compensating for larger unexpected errors or perturbations.

### SimpleDIVA

While the full DIVA model provides a comprehensive account of speech motor control, direct application of the model to experimental and clinical datasets requires a simplified implementation. To address this need, Kearney et al. (2020) developed SimpleDIVA, a three-parameter mathematical model derived from the DIVA framework. SimpleDIVA decomposes the full model into its three core control subsystems, providing parameter estimates for auditory feedback control (αA), somatosensory feedback control (αS), and feedforward control/learning rate (λFF). Given data from an auditory perturbation experiment, SimpleDIVA identifies optimal values for these parameters, yielding a mechanistic interpretation of the speaker’s behaviour that is not readily available from the behavioural data alone.

SimpleDIVA provides excellent fits to perturbation data across different acoustic dimensions (formant frequencies, fundamental frequency) and experimental paradigms (Kearney et al., 2020). Importantly, the three-parameter structure avoids the parameter redundancies often present in more complex models (including the full DIVA model), which enables unique and meaningful fits for individual participants. This property makes SimpleDIVA a potentially valuable clinical tool. That is, by comparing a patient’s parameter values to normative distributions, clinicians and researchers may be able to characterise the relative integrity of each control subsystem. For example, a patient with a reduced auditory feedback gain parameter but preserved feedforward learning rate parameter would present a different mechanistic profile, and may warrant a different therapeutic approach, than one with the reverse pattern. SimpleDIVA is freely available as a standalone application (requiring no MATLAB licence), making it accessible for both clinical and research use.

### Simulating a haemodynamic response

**Figure 3.**
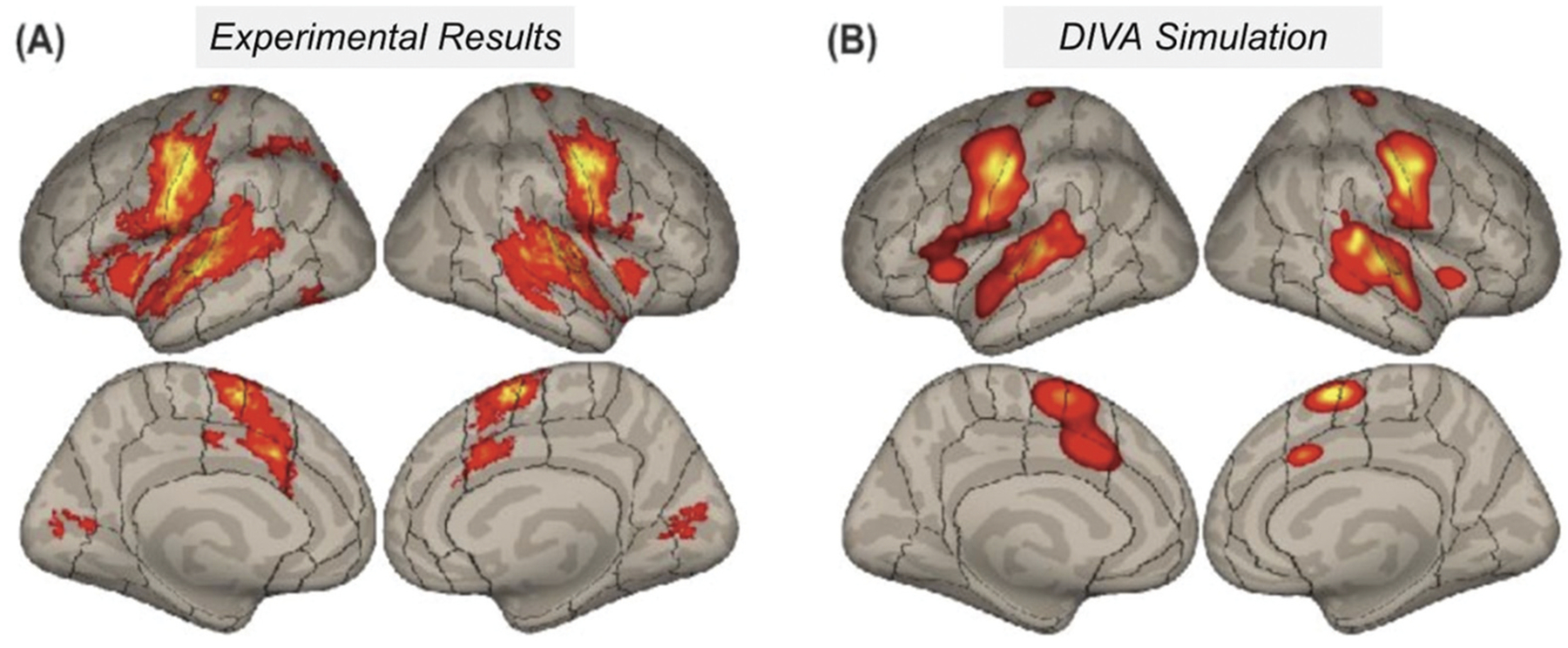
(A) Brain activity measured with functional magnetic resonance imaging (fMRI) while subjects read syllables aloud, contrasted with a baseline involving passive viewing of letters plotted on inflated cortical surfaces. Top panels show left and right lateral views; bottom panels show left and right medial views. (B) Simulated fMRI activations derived from the DIVA model’s node activities during syllable production. Adapted with permission from (Guenther, 2016).

Beyond serving as a theoretical framework, the DIVA model is instantiated as a neural network whose architecture mirrors the human speech network. It includes both cortical areas and subcortical structures such as the basal ganglia and cerebellum. The basal ganglia and thalamus contribute to the initiation and selection of speech movements (Alexander et al., 1986; Alm, 2004; Chang & Guenther, 2020), while cerebellar loops modulate cortical motor output to fine-tune the timing and coordination of these movements (Holmes, 1917). Each model component is associated with a specific brain region, and during simulations, it generates an electrical response (akin to neuron spiking) and a haemodynamic response (akin to blood flow measured by functional magnetic resonance imaging). These simulated whole-brain activity maps allow researchers to compare predicted neural activation patterns directly with those observed in empirical studies (Figure 3), providing a powerful means of validating the model and grounding it in biological reality.

## Part II: Speech disorders and the DIVA model

**Figure 4.**
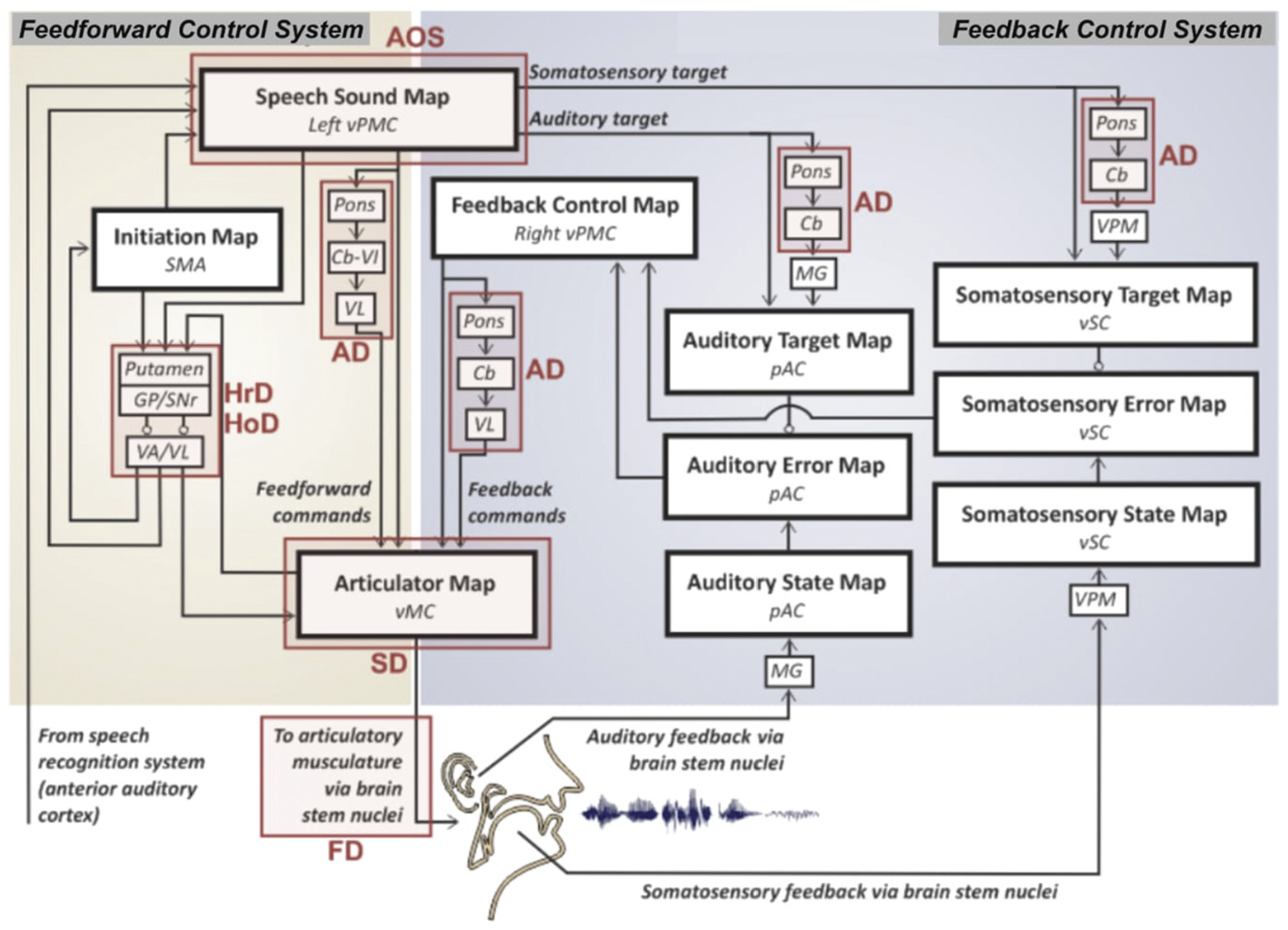
Sites of neural impairment (red boxes) associated with various speech disorders (red text labels) within the context of the DIVA model. These disorders include flaccid dysarthria (FD), spastic dysarthria (SD), ataxic dysarthria (AD), hyperkinetic dysarthria (HrD), hypokinetic dysarthria (HoD), apraxia of speech (AOS), and supplementary motor area syndrome (SMAS). Cb, cerebellum (specific lobule unknown); Cb-VI, cerebellum lobule VI; GP, globus pallidus; MG, medial geniculate nucleus of the thalamus; pAC, posterior auditory cortex; SMA, supplementary motor area; SNr, substantia nigra pars reticula; VA, ventral anterior nucleus of the thalamus; VL, ventral lateral nucleus of the thalamus; vMC, ventral motor cortex; VPM, ventral posterior medial nucleus of the thalamus; vPMC, ventral premotor cortex; vSC, ventral somatosensory cortex. Adapted with permission from Guenther (2016).

A major strength of the DIVA model lies in its ability to bridge from theoretical neuroscience to clinical relevance. As a neurocomputational model grounded in anatomical and functional brain data, the DIVA model provides a principled way to identify which neural circuits are involved in different components of speech production (Figure 4). This makes it a powerful tool for understanding communication disorders and their neural bases. By simulating ‘damaged’ versions of the model, such as removing or altering the function of specific circuits, researchers can explore which patterns of impairment correspond most closely to those observed in clinical populations. These simulations have already begun to shed light on disorders such as apraxia of speech, stuttering, and dysarthrias associated with cerebellar or basal ganglia dysfunction (e.g. Civier et al., 2013; Terband et al., 2009).

### Relative importance of feedforward and feedback systems

One important insight demonstrated by the model is the developmental shift in the relative roles of feedforward and feedback control systems (Guenther et al., 2006). In mature, fluent speech, motor programs generated by the feedforward control system serve as the primary driver of articulatory commands (cf. Schmidt, 1975; Houde & Jordan, 2002; Kawato, 1990, Guenther et al., 2006; Tourville & Guenther, 2011). The auditory and somatosensory feedback systems play a more limited role, primarily engaged when unexpected perturbations or errors occur (Abbs, 1973). The adult speaker’s ability to speak intelligibly even in the absence of auditory feedback (e.g. in noisy environments or after hearing loss) further highlights this reliance on feedforward control (Cowie & Douglas-Cowie, 1992).

In contrast, for children learning to speak, feedback control mechanisms are essential. Auditory feedback plays a key role in shaping speech motor programs during early development and in maintaining accuracy as the vocal tract changes with growth (e.g. Sharkey & Folkins, 1985; Smith & Goffman, 1998; Smith & Zelaznik, 2004). Damage to the auditory feedback control system in children can therefore be especially disruptive to the learning of speech motor plans, but it does not cause major articulation anomalies in adults (e.g. Stoel-Gammon & Otomo, 1986). In contrast, significant damage to the feedforward control system at any age will force an over-reliance on ill-suited feedback control mechanisms (e.g. Parrell et al., 2017) and have profound effects on speech, as this system is essential for generating well-coordinated motor commands (Guenther, 2016).

### Somatosensory processing in the corticobulbar system

An important and often overlooked implication of the DIVA model concerns the distinct role of somatosensory processing within the speech motor system. Most somatosensory research in neurology has focused on limb and corticospinal function (e.g. Borich et al., 2015), whereas a small but growing literature examines sensory contributions to speech and voice control (e.g. laryngeal somatosensory and speech sensorimotor adaptation in PD; Mollaei et al., 2013). The DIVA model makes clear that somatosensory feedback plays a critical and distinct role in speech motor control via the corticobulbar system, which carries descending projections from cortex to the brainstem motor nuclei innervating the muscles of the face, jaw, tongue, velum, pharynx, and larynx. This speech-specific somatosensory circuit is related to, but functionally distinct from, the somatosensory pathways serving limb motor control. The articulators are among the most densely innervated structures in the body, with extensive cortical representation in both motor and somatosensory cortex (Penfield & Boldrey, 1937). Speech requires very small, rapid adjustments of the tongue, lips, and velum, imposing unique demands on somatosensory processing that exceed those needed for limb control (Nasir & Ostry, 2006; Tremblay et al., 2003). Moreover, because many speech articulators (e.g. the velum, pharyngeal walls, larynx) lack visual feedback channels, somatosensory information may serve as the primary non-auditory sensory source available for monitoring and correcting speech production.

This distinction between speech and limb somatosensory processing has important clinical implications across multiple speech motor disorders. In ataxic dysarthria, cerebellar damage is associated with abnormalities in limb sensorimotor integration and somatosensory processing (Schmahmann, 2004). The DIVA model predicts that cerebellar damage may also degrade the reliability of somatosensory speech targets, potentially contributing to the articulatory variability that characterises this disorder beyond what can be attributed to impaired feedforward timing alone. In hypokinetic dysarthria associated with Parkinson’s disease, there is growing evidence that somatosensory processing is impaired alongside motor initiation deficits (Conte et al., 2013). Within the DIVA framework, such somatosensory dysfunction is expected to compound the speech deficit, as degraded proprioceptive monitoring of the articulators may limit the system’s ability to detect and correct the reduced articulatory excursions characteristic of parkinsonian speech (Ackermann & Ziegler, 1991). In hyperkinetic dysarthria, aberrant sensorimotor integration and inhibitory control within the basal ganglia circuit may contribute to the failure to appropriately inhibit involuntary movements (Hallett, 2011). Taken together, these observations underscore that assessments and interventions for speech motor disorders should account for the unique demands of the corticobulbar system, rather than assuming that patterns observed in limb function will generalise to speech.

### Initiation versus articulation

The feedforward system in DIVA can itself be divided into two functionally distinct corticosubcortical circuits: one for initiation and one for articulation. The articulation circuit, which heavily involves ventral premotor and motor cortices and the cerebellum, generates the complex muscle activation patterns that move the articulators and establish motor programs. The initiation circuit, which heavily involves the supplementary motor area (SMA) and basal ganglia, is responsible for activating (initiating) and de-activating (terminating) these motor programs at the appropriate times.

An analogy for these circuits can be drawn to a jukebox. A jukebox has two key subsystems. The first decides which record to play next and when to start it. This ‘selection and timing’ function is analogous to the initiation circuit in speech, which relies centrally on cortico-basal ganglia loops to trigger motor programs at the right moment. The second subsystem is the mechanism that physically plays the record, converting grooves on vinyl (or data on a CD) into music. This is analogous to the articulation circuit, where corticocerebellar loops generate and coordinate the detailed muscle movements that produce speech sounds.

Just as a jukebox needs both parts to work smoothly – the selector to choose and cue up songs, and the player to translate data on disc into acoustic signals – speech production requires both the initiation system to time the onset/offset of motor programs and the articulation system to execute them with precision. If the selector malfunctions, the song may not start, start late, or repeat inappropriately; if the player malfunctions, the chosen song will sound distorted. Similarly, disruptions in initiation circuits can cause problems with starting and stopping speech, while disruptions in articulation circuits can impair the accuracy and fluidity of speech movements.

### Disorders of articulation: Ataxic dysarthria

This distinction between articulation and initiation becomes especially relevant when considering different speech motor disorders. Ataxic dysarthria is a disorder of articulation most commonly associated with cerebellar damage. According to the DIVA model, the cerebellum plays a central role in the articulation circuit of the feedforward control system. It is responsible for generating the finely tuned, precisely timed motor commands required for smooth and coordinated articulatory movements (Ackermann & Hertrich, 2000). Damage to this region, as seen in individuals with ataxic dysarthria, disrupts the accuracy and stability of these motor programs (Ackermann et al., 2007; Spencer & Slocomb, 2007). The result is the hallmark ‘scanning’ speech pattern of ataxia, which is characterised by imprecise consonant and vowel production, irregular articulatory breakdowns, and disrupted prosody due to poorly coordinated timing and force of articulator movements (Darley et al., 1969; Duffy, 2013; Hilger et al., 2023; Schalling & Hartelius, 2013).

Within the DIVA framework, this disruption is understood primarily as a breakdown in feedforward control. While the feedback control subsystems (auditory and somatosensory) may remain structurally intact in individuals with cerebellar damage, they are typically unable to compensate in real time for the degraded feedforward commands (e.g. Parrell et al., 2017). This is especially true during fluent speech, where the temporal constraints of communication far outpace the relatively slow processing loops of sensory feedback correction. As such, despite the presence of functional feedback systems, the degraded feedforward commands dominate speech production and result in the characteristic impairments.

The DIVA model also generates predictions beyond the primary deficit in articulation. Specifically, it hypothesises that cerebellar damage may lead to more subtle, secondary impairments in the development or stability of auditory and somatosensory speech targets. Although these deficits remain largely untested, they would imply that sensory feedback control is also at least mildly compromised – not due to a failure in error detection or correction per se, but because the sensory reference targets themselves are less reliable. These impairments are unlikely to be apparent in everyday speech, but may become evident in experiments that involve learning novel speech patterns or responding to subtle perturbations in auditory or somatosensory feedback timing or content. Such experimental paradigms could reveal the extent to which cerebellar damage affects not only the production of motor commands but also the fidelity of sensory representations that guide feedback-based learning.

Clinically, this framework helps explain why individuals with ataxic dysarthria often exhibit speech disturbances marked by irregular articulatory breakdowns, fluctuating consonant and vowel precision, and disrupted prosody. Within the context of the DIVA model, this variability reflects the breakdown of cerebellar timing and coordination within the articulation circuit, leading to unstable feedforward commands that cannot be adequately corrected by feedback systems. For clinicians, this has important treatment implications, highlighting the value of strategies that reduce speech rate, enhance prosodic control, and improve articulatory precision through rhythm and pacing techniques. These approaches can be understood as ways of externally stabilising speech timing to partially compensate for the unreliable internal timing mechanisms disrupted by cerebellar damage.

### Disorders of articulation: Flaccid dysarthria

Some speech motor disorders affect a still more downstream component: the final common pathway through which all motor commands are ultimately executed. Flaccid dysarthria exemplifies this category. Flaccid dysarthria results from damage to lower motor neurons (LMNs), including the cranial nerve nuclei in the brainstem and the peripheral nerves that innervate the muscles of speech (Duffy, 2013). The nerves that are involved thus determine which articulators are affected (Langmore & Lehman, 1994). Within the DIVA model, this corresponds to disruption of the pathway downstream of the ventral motor cortex, where neural commands are converted into muscle activations that move the articulators. Because all motor commands must pass through this pathway, damage at this level disrupts the execution of both feedforward and feedback-driven commands indiscriminately. The resulting speech characteristics reflect the specific muscles affected and the nature of the LMN damage. Depending on which cranial nerves are involved, individuals with flaccid dysarthria may exhibit breathy or hoarse voice quality (cranial nerve X), hypernasality (cranial nerve X, pharyngeal branch), imprecise articulation (cranial nerves V, VII, XII), or combinations of these features (Darley et al., 1969; Duffy, 2013). A distinguishing characteristic of flaccid dysarthria is that the impairment tends to be consistent and predictable, reflecting the direct relationship between denervation and muscle weakness (Duffy, 2013). This contrasts with the variable or context-dependent errors seen in disorders affecting higher-level motor programming (Kent & Rosenbek, 1983; Strand et al., 2014).

From the perspective of the DIVA model, the feedforward motor programs and feedback control targets may remain intact at the cortical level in flaccid dysarthria, but the motor system’s ability to faithfully execute these commands is degraded. This is consistent with established clinical practice, in which intervention typically focuses on strengthening residual muscle function (Yorkston, 2010) or compensating for structural deficits (e.g. palatal prostheses for velopharyngeal incompetence), rather than on approaches targeting motor programming or initiation.

### Disorders of articulation: Spastic dysarthria

Spastic dysarthria results from bilateral damage to the upper motor neurons (UMNs) that project from the cortex to the brainstem motor nuclei via the corticobulbar tract (Duffy, 2013). UMN damage is associated with muscle spasticity, which may result in slow and laboured speech (Darley et al., 1969; Langmore & Lehman, 1994). Unlike flaccid dysarthria, in which the final common pathway is directly compromised, spastic dysarthria involves impaired modulation of that pathway. Specifically, it involves a loss of the inhibitory control that normally regulates the excitability of lower motor neurons.

Within the DIVA model, this maps onto a disruption of the descending projections from the ventral motor cortex and associated cortical regions to the brainstem nuclei. Because the corticobulbar tract carries both direct motor commands and modulatory signals that regulate muscle tone and reflex activity, bilateral damage leads to hypertonicity, hyperreflexia, and reduced range of movement in the speech musculature. This results in excessive and poorly graded force that impairs the smooth, rapid movements required for speech (Duffy, 2013). The speech characteristics of spastic dysarthria typically include a slow rate, strained-strangled voice quality, reduced pitch and loudness variation, hypernasality, and imprecise but consistent articulation errors (Darley et al., 1969; Duffy, 2013). The feedforward motor programs generated at the cortical level may be appropriately specified, but the corticobulbar pathway’s ability to transmit and modulate these commands is compromised. Clinically, understanding spastic dysarthria within this framework highlights why intervention often focuses on strategies to reduce effort, improve phonatory control, and optimise articulatory precision within the constraints of increased muscle tone.

### Disorders of articulation: Apraxia of speech

Apraxia of speech (AOS) is a disorder of articulation characterised by impaired planning and sequencing of speech movements, despite intact muscle strength and basic motor execution. Individuals with AOS often exhibit distorted sound substitutions, inconsistent errors, and visible groping behaviours as they struggle to initiate or coordinate the motor plans needed for fluent speech (Duffy, 2013; Strand et al., 2014). The DIVA model accounts for these disruptions as stemming from damage to the speech sound map located in the left ventral premotor cortex, a region critical for the generation and retrieval of feedforward motor commands (see Miller & Guenther, 2021 for a review).

In typical speech, the speech sound map stores the learned motor programs that specify the time-varying articulatory movements required to produce each syllable or word. These feedforward commands allow for efficient, pre-planned articulation without continuous reliance on sensory feedback. However, in AOS, damage to this map degrades or erases these stored motor programs, forcing speakers to compensate with incomplete or incorrect articulatory sequences (Kent & Rosenbek, 1983; Odell & Shriberg, 2001). The resulting speech may be effortful, halting, and imprecise (Darley et al., 1969), reflecting the breakdown of feedforward control.

Beyond this core impairment, the DIVA model also predicts a secondary consequence: compromised feedback-based speech regulation. While the auditory and somatosensory feedback systems themselves may remain structurally intact, their effectiveness depends on accessing accurate sensory targets (i.e. internal representations of what speech should sound and feel like). Because these target projections originate (in part) from the speech sound map, damage to this region may limit the brain’s ability to detect and correct speech errors in real time (Bislick et al., 2017). This hypothesis, though not yet empirically tested, may help explain why some individuals with AOS struggle to benefit from feedback-driven cues or interventions.

Clinically, this dual deficit helps account for the variable and often treatment-resistant nature of AOS. Unlike dysarthria, where consistent distortions can sometimes be compensated with clear strategies or slowed rate (Tjaden et al., 2014), AOS is marked by inconsistency, failed initiation attempts, and trial-and-error groping (Duffy, 2013; Strand et al., 2014). Within the context of the DIVA model, this variability reflects the absence of stable feedforward programs to rely on, paired with weakened ability to use sensory feedback as a backup system. This also provides insight into why therapy for AOS often emphasises repetitive practice of specific speech sounds or syllables; through massed practice, clinicians aim to help the brain re-establish more reliable feedforward commands and strengthen the mapping between auditory/somatosensory targets and articulatory movements (Maas et al., 2008). Emerging neuromodulation and motor-learning – based approaches may similarly leverage the plasticity of the speech sound map to restore more consistent motor planning capacity.

### Disorders of initiation: Hypokinetic dysarthria

Initiating and scaling speech motor programs requires precise coordination within a neural circuit that centrally involves the basal ganglia (Groenewegen, 2003). In the DIVA model, the initiation circuit is responsible for determining both when a speech motor program is launched and how intensely it is executed. It functions by regulating the balance between two main pathways: the excitatory direct pathway and the inhibitory indirect pathway. Together, these allow the brain to dynamically select, initiate, and modulate feedforward motor commands necessary for fluent speech.

Disruptions to this circuit can impair a speaker’s ability to initiate movement (e.g. blocks in stuttering) (Middleton & Strick, 2000), attenuate the size or ‘vigor’ of movements (e.g. hypophonia and reduced emphatic stress in Parkinson’s disease; Turner & Desmurget, 2010), or trigger unintended movements such as chorea in Huntington’s disease (Mink, 2003) or muscle hyperactivity in laryngeal dystonia (Simonyan et al., 2017). Such disruptions underlie a number of clinical communication disorders, all of which fall under the broader category of *disorders of initiation* in the DIVA framework. A prominent example is hypokinetic dysarthria, which is commonly observed in individuals with Parkinson’s disease (PD). In PD, the gradual loss of dopaminergic neurons in the substantia nigra leads to a shift in the balance of the initiation circuit, such that the inhibitory indirect pathway becomes overly dominant (Bartels & Leenders, 2009). This imbalance dampens the initiation of motor programs and reduces the force with which these programs are executed.

From a DIVA perspective, this pathological shift means that feedforward motor commands for speech are initiated with less consistency and reduced intensity. The result is speech that is often described as quiet, monotone, breathy, or lacking in articulatory precision, which are all classic hallmarks of hypokinetic dysarthria (Darley et al., 1969; Ramig et al., 2011; Tjaden, 2008). Importantly, these impairments do not arise from breakdowns in the articulation of speech per se, but rather from a compromised ability to effectively initiate and scale motor commands that have otherwise been correctly learned.

Clinically, viewing hypokinetic dysarthria through this initiation-circuit lens underscores why patients with Parkinson’s disease often present with reduced loudness, limited pitch variability, and short rushes of speech. The DIVA model provides a valuable framework for understanding these dynamics, emphasising that the speech deficits seen in hypokinetic dysarthria are not simply mechanical in nature but rooted in neural timing and intensity regulation. By simulating this imbalance in the cortico-basal ganglia initiation circuit, the model can replicate the diminished vocal effort and hesitations characteristic of PD-related speech. Moreover, it helps clarify why speech therapy strategies that increase vocal amplitude (e.g. Lee Silverman Voice Treatment; Ramig, 2001) are often effective; they may temporarily recalibrate the impaired initiation signals and ‘override’ the underactive feedforward command.

### Disorders of initiation: Hyperkinetic dysarthria

Whereas hypokinetic dysarthria results from diminished activation of motor programs due to an overactive inhibitory pathway in the basal ganglia, hyp*er*kinetic dysarthria emerges from the opposite imbalance: excessive activation of motor programs. Within the initiation circuit, the excitatory direct pathway facilitates movement initiation, while the inhibitory indirect pathway acts as a counterbalance, suppressing competing or inappropriate motor commands.

In hyperkinetic dysarthria, as seen in conditions like Huntington’s disease, this balance is disrupted such that the direct pathway becomes overly dominant, overriding the modulatory influence of the indirect pathway (Albin et al., 1989). As a result, speech motor programs are initiated erratically or inappropriately, producing involuntary, excessive, and poorly controlled vocal movements (Barkmeier-Kraemer & Clark, 2017; Darley et al., 1969). These can include unpredictable changes in pitch, loudness, and articulation, often leading to speech that is perceived as strained, jerky, or effortful (Darley et al., 1969). The DIVA model provides a neurocomputational framework for understanding this phenomenon. When the inhibition normally provided by the indirect pathway is reduced or absent, the model predicts frequent initiation of motor programs that were not intended or optimally timed. This leads to what could be conceptualised as ‘false positives’ in speech initiation – motor gestures are triggered too often or in the wrong context. These involuntary activations can interrupt ongoing speech or layer additional movement atop intended gestures, degrading speech intelligibility and fluency.

Another disorder involving impaired processing in the initiation circuit is laryngeal dystonia, a condition characterised by excessive muscle activity in the vocal folds, resulting in strained or spasmodic voice quality (Blitzer et al., 2018; Phukan et al., 2011). While the exact aetiology of laryngeal dystonia is still under investigation, it is hypothesised that an imbalance favouring the excitatory direct pathway in the basal ganglia loop could lead to inappropriate activation of laryngeal musculature (Hallett, 2011; Simonyan et al., 2017).

Clinically, the DIVA framework provides a rationale for why individuals with hyperkinetic dysarthria often present with unpredictable pitch breaks, sudden changes in loudness, and involuntary articulatory movements that disrupt fluency. These symptoms do not reflect weakness of the speech muscles but rather excessive and poorly regulated initiation of motor commands. From a treatment standpoint, this distinction is important; behavioural therapies typically focus on strategies to stabilise respiratory and phonatory support (Barkmeier-Kraemer & Clark, 2017), while in some cases pharmacologic or neuromodulatory interventions are used to dampen excessive basal ganglia output (Jankovic, 2009). By modelling hyperactivation in the initiation circuit, the DIVA framework helps explain the erratic nature of hyperkinetic speech disorders. It also clarifies why interventions that reduce involuntary motor output – whether through medication, botulinum toxin injections (as in laryngeal dystonia), or compensatory speaking strategies – can improve speech intelligibility and communicative effectiveness.

### Disorders of initiation: Stuttering

Stuttering is a speech disorder characterised by involuntary disruptions in fluency, such as sound or syllable repetitions, prolongations, and blocks predominantly occurring at the onset of words or syllables (Guitar, 2019). Unlike purely articulatory disorders, stuttering is increasingly understood as a disruption in the initiation of speech motor programs, rather than in the formation or execution of the programs themselves (Alm, 2004; Guenther, 2016; Peters & Hulstijn, 1987).

Within the DIVA model framework, stuttering is conceptualised as a system-level impairment of the cortico-basal ganglia initiation circuit, which is responsible for triggering and timing motor program execution. In this view, the core deficit lies in the unstable or mistimed initiation signals that originate from the basal ganglia and supplementary motor area (see also Alm, 2004). When this system fails to deliver consistent, well-timed activation to the speech motor cortex, the result is a delay, misfire, or repetition of the motor program. The speaker may attempt to initiate a word, but the command either fails to launch or becomes trapped in a loop of partial or hesitant execution.

A growing body of neuroimaging and neurophysiological studies supports this interpretation. For instance, stuttering has been associated with reduced structural and functional connectivity in left hemisphere speech motor areas (e.g. Cai et al., 2014; Chang & Zhu, 2013; Chow & Chang, 2017; Korzeczek et al., 2021; Sommer et al., 2002; Watkins et al., 2008), which could impair the timing and efficiency of initiation signals. Additionally, anatomical and functional anomalies in left premotor and motor cortex have been reported in people who stutter (e.g. Garnett et al., 2018), potentially undermining the reliable execution of feedforward motor plans. Increased structural connectivity in right hemisphere frontal regions has also been found to scale with stuttering severity (Neef et al., 2018). Behavioural evidence has similarly indicated that children who stutter exhibit deficits in both speech motor planning and execution (Walsh et al., 2015), consistent with systems-level impairments in the circuits supporting speech motor sequencing.

The basal ganglia itself is another likely contributor (Alm, 2004). Studies have suggested abnormal dopaminergic signalling in stuttering, with excess dopamine levels potentially disrupting the balance between the excitatory direct and inhibitory indirect pathways of the initiation loop (e.g. Wu et al., 1997). This imbalance could lead to unstable thresholds for triggering motor programs – sometimes failing to initiate speech and other times launching unwanted repetitions or prolongations.

Simulations of these hypotheses have been implemented in computational models based on DIVA, such as those developed by Civier et al. (2013), which replicate many of the timing disruptions observed in individuals who stutter. By selectively altering parameters related to basal ganglia output, hemispheric connectivity, and cortical activation thresholds, these models demonstrate how disruptions at different levels of the initiation circuit can produce the characteristic disfluencies of stuttering. This conceptualization is also consistent with broader proposals that stuttering may be best understood as a spectrum disorder involving multiple interacting neural and behavioral phenotypes rather than a single unitary deficit (SheikhBahaei et al., 2023).

Clinically, framing stuttering as a disorder of initiation rather than articulation helps explain why individuals who stutter often report a sense of being ‘stuck’ or blocked at the onset of speech, despite knowing exactly what they want to say and having the motor capacity to produce it. The repetitions, prolongations, and blocks that define stuttering can be understood as outward signs of unstable or mistimed initiation signals in the basal ganglia – SMA loop. This perspective highlights why fluency often improves under conditions that alter timing demands (e.g. slowed speech, choral reading, or singing) as these reduce the burden on the initiation circuit and provide external cues to stabilise motor program triggering (Andrews et al., 1982; Max et al., 2004). It also underscores the rationale for therapies that target initiation processes directly, whether through behavioural timing strategies (Park & Logan, 2015), rhythm- or pacing-based techniques (Brady, 1969), or pharmacological interventions aimed at modulating dopaminergic activity (Maguire et al., 2020). By situating stuttering within the initiation framework of the DIVA model, clinicians gain a mechanistic explanation for both its hallmark symptoms and the contexts in which fluency can be facilitated.

The DIVA model’s account of stuttering as a disorder of initiation provides a compelling framework for understanding the core motor deficit, but it does not directly address the well-documented influence of phonological and linguistic factors on stuttering behaviour. For example, stuttering frequency is known to be affected by phonological complexity, word position within utterances, and the phonotactic probability of syllable sequences (e.g. Bernstein Ratner, 1997; Smith et al., 2010; Yaruss, 1999).

To account for these influences, it is helpful to consider the GODIVA (Gradient Order DIVA) model, an extension of DIVA that models the phonological encoding and sequential planning of speech (Bohland et al., 2010). GODIVA extends the DIVA framework by adding two functionally distinct cortico-basal ganglia loops: a planning loop responsible for phonological working memory and sequential ordering of upcoming speech sounds, and a motor loop involved in the generation and initiation of motor commands for the current speech sound (Bohland et al., 2010; Civier et al., 2013). Emerging empirical work supports this dual-loop distinction and suggests that the planning and motor circuits may be differentially affected across individuals who stutter. Rowe et al. (2024) found preliminary evidence of planning and motor subtypes based on resting-state functional connectivity in adults who stutter, while Höbler et al. (2026) found reduced within-network density in both the planning and motor loops among children who stutter.

This dual-loop architecture also provides a framework for understanding how phonological factors may influence stuttering. When phonological complexity increases, for example in utterances with longer or less frequent syllable sequences, greater demands are placed on the planning loop, potentially delaying the activation of speech sound map nodes that carry out speech motor programs. If the activation of the intended syllable does not reach the selection threshold in a timely manner, the result may be a stuttered disfluency (e.g. a block if the activation stalls, a repetition if the selection signal oscillates, or a prolongation if the system attempts to maintain an ongoing gesture while awaiting the next command; Civier et al., 2013). Indeed, simulations of the GODIVA model have demonstrated how excess dopamine can delay syllable selection for word-initial syllables, while deficient white matter connectivity can delay selection for non-initial syllables, each producing distinct types of disfluency that map onto patterns observed in individuals who stutter (Civier et al., 2013).

The prefrontal regions implicated in GODIVA’s planning loop, particularly the inferior frontal sulcus and pre-supplementary motor area, overlap substantially with regions associated with executive control functions, including working memory, cognitive flexibility, and inhibitory control (Aron et al., 2014; Badre, 2008; Koechlin & Summerfield, 2007). This overlap may provide a neuroanatomical basis for understanding how executive functioning difficulties, which have been documented in some individuals who stutter (e.g. Eggers et al., 2013; Ntourou et al., 2013), could interact with speech motor planning processes. Specifically, if the prefrontal networks supporting phonological sequence planning are also recruited for domain-general executive functions, then competing demands on these shared resources could further destabilise the planning loop and increase the likelihood of disfluency.

### Limitations and open questions

The DIVA model provides a powerful framework for understanding speech motor control and its disorders. However, several limitations warrant acknowledgement. First, DIVA is fundamentally a model of motor programming and execution that does not address upstream linguistic processes such as lexical access or phonological encoding. The GODIVA model (Bohland et al., 2010) extends the framework to address phonological encoding and sequential planning, but even the combined DIVA/GODIVA system does not model the full chain from conceptual preparation to articulation. This means that clinical phenomena arising from linguistic-motor interactions are only partially accounted for. Second, the model does not fully formalise the role of somatosensory processing during speech development and its transition to a more limited role in adult production. Thus, the conditions under which somatosensory targets are updated remain an open question (see Guenther, 2016 for discussion). Third, while the model’s architecture can be extended to longer utterances, much of the computational validation to date has focused on single-syllable productions. Capturing the full dynamics of connected speech (e.g. prosodic variation and speaking rate effects) remains an area for continued development. In addition, several model predictions discussed in this paper, such as hypothesised secondary impairments in sensory targets following cerebellar or speech sound map damage, remain empirically untested and represent important directions for future research.

## Conclusions

The DIVA model provides a powerful framework for understanding both the mechanisms of speech production and their disruption in communication disorders. Beyond serving as a theoretical map, DIVA functions as a diagnostic and predictive tool: its ability to simulate neural damage, anticipate resulting deficits, and map symptoms to specific control systems offers a pathway to personalised, mechanistically informed treatment strategies. In the longer term, as our understanding of speech-related neural circuits advances, we aim to leverage this pathway to guide the development of more targeted and effective therapies for individuals living with speech motor disorders (see also Chang et al., 2025).

## Data Availability

Data sharing is not applicable to this article as no new data were created or analysed in this study.
